# Supplementary figures and images for: Live Virus Neutralisation of the 501Y.V1 and 501Y.V2 SARS-CoV-2 Variants following INO-4800 Vaccination of Ferrets
Source: Front Immunol. 2021 Jun 25;12:694857. doi: 10.3389/fimmu.2021.694857 (PMC8269317; doi:10.3389/fimmu.2021.694857)

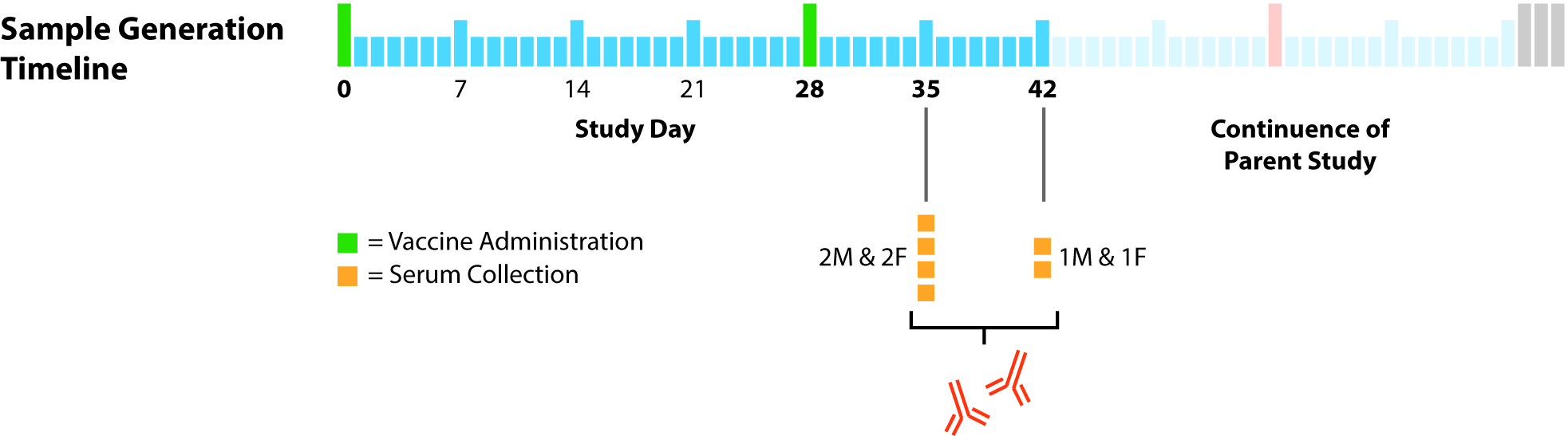

Supplement: Supplementary Figure 1 — Outline of serum sample generation timeline. Ferrets (n=3 male, n=3 female) were vaccinated with INO-4800 on Day 0 and Day 28 of the study (green dates). Serum samples used for neutralisation assays were collected on Day 35 (n=2 each from male and female) or 42 (n=1 each from male and female) of the study. The parent study continued beyond Day 42, and is not described in this manuscript. [file Image_1.tif]

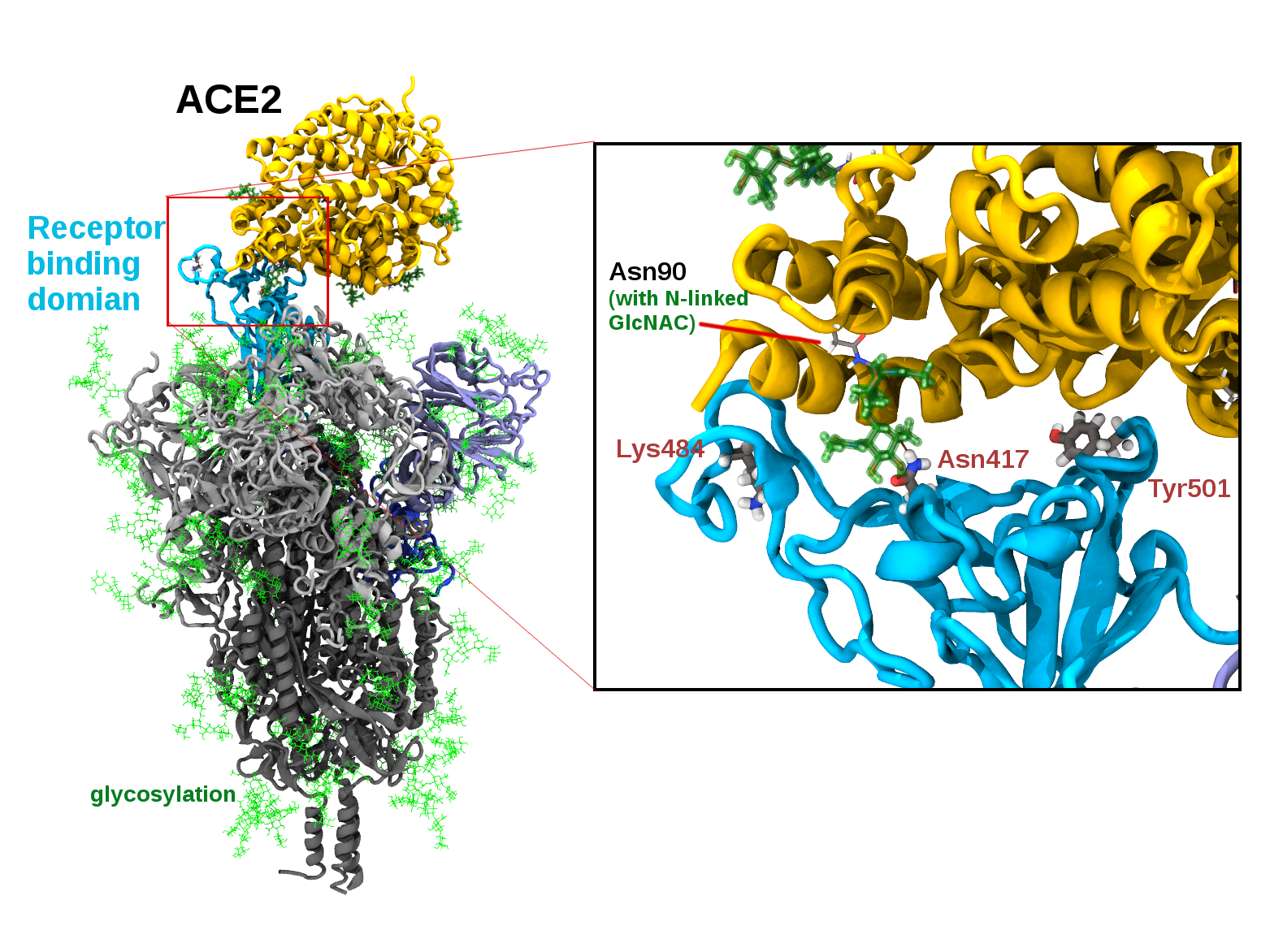

Supplement: Supplementary Figure 2 — Structure of ACE2 binding to the SARS-CoV-2 Spike receptor-binding domain of variant 501Y.V2. Modelling suggests the lysine to asparagine mutation, (K417N), may facilitate additional hydrogen bonding to the ACE2 receptor, (Angiotensin-converting enzyme 2), via binding through an N-linked glycoside at position asparagine Asn90 in ACE2. [In this case modelled as Beta-D-GlcNAC (1->4) GlcNAC]. The relative position of the other 501Y.V2 mutations in the receptor-binding domain (Tyr501 and Lys484) as also shown. [file Image_2.tiff]

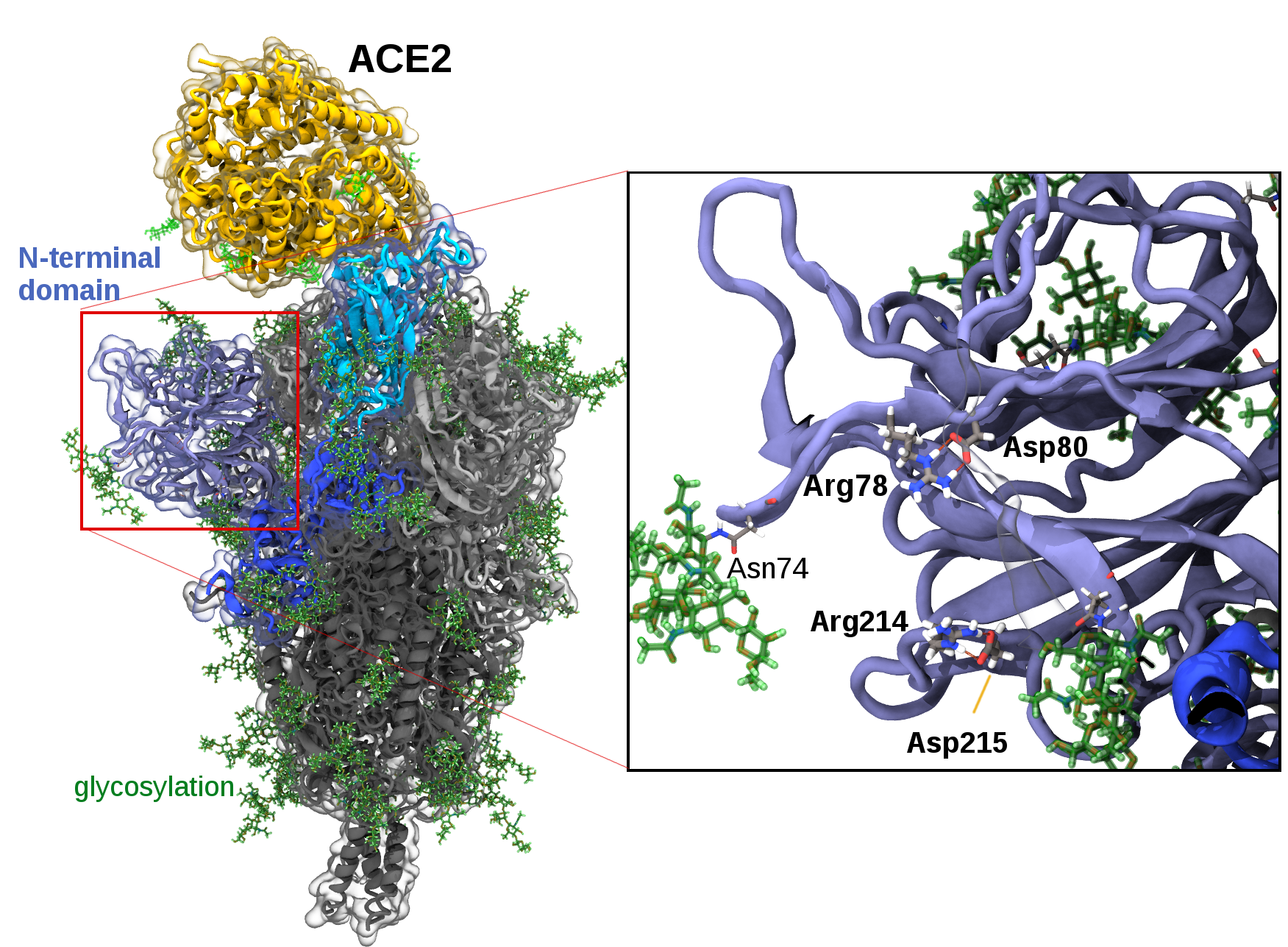

Supplement: Supplementary Figure 3 — Structure of ACE2 binding to the SARS-CoV-2 Spike receptor-binding domain showing a close up of the N-terminal domain region, highlighting the salt bridges between arginine and aspartic acid at positions Arg78-Asp80 and Arg214-Asp215. In the 501Y.V2 variant, aspartic acid 80 is mutated to alanine (D80A) and aspartic acid 215 is mutated to glycine (D215G), losing both salt bridges, altering the relative positions of the arginine residues, as shown in Figure 2E ). The asparagine N-linked glycosylation attachment point Asn74 is also labelled in the diagram. [file Image_3.tiff]
